# Case of Cynanche Cellularis, with Remarks

**Published:** 1822-10

**Authors:** George Gregory

**Affiliations:** Physician to the Small-Pox Hospital, and One of the Physicians to the St. George's and St. James's General Dispensary.


Art. II.—*Case of Cynanche Cellularis, with Remarks.* By George Gregory, M.D. Physician to the Small-Pox Hospital, and one of the Physicians to the St. George's and St. James's General Dispensary.

The case which I have now the honour of laying before the readers of the London Medical and Physical Journal, is, I have reason to believe, extremely rare. It has never occurred to myself to witness any thing at all similar to it, either before or since; nor have I been able to ascertain that, in the practice of any of my professional friends, an analogous case has ever presented itself. The disease consisted in an extensive inflammation of the cellular membrane of the neck and anterior mediastinum, of a highly malignant character. Its course was rapid, and the symptoms which attended it were of unusual severity. It bore, in the first instance, the appearance of a rheumatic affection of the joints of the cervical vertebræ. At a somewhat later period of the disease, it was imagined that the thyroid gland was the immediate seat of inflammation; but it was not until after death that the exact nature of the case was understood. Books have afforded me but very scanty information concerning this affection; but, before alluding in a particular manner to the few cases which I have met with that appear to resemble it in some of its pathological features, it will be right to give the details of that which furnishes the subject of the present memoir.

Ann Jones, twenty-five years of age, housemaid, was attacked on Tuesday, February 13, 1821, with feverish symptoms and pains of the back part of the neck, resembling rheumatism. She was not conscious of having exposed herself in any particular manner to cold. For these complaints she was bled, the same evening, to the extent of a pint, and took opening physic. The following day she came under my care; and at two o'clock P.M., when I first saw her, the following was the state of her symptoms.

She had a considerable degree of fever, attended with great difficulty of swallowing. There was swelling, hardness, and some tenderness of the external parts of the throat. The swelling and tenderness extended round the neck on each side, but were chiefly felt and complained of at the junction of the clavicles with the sternum. On inspection of the internal fauces, no enlargement of the tonsils, or redness, or ulceration of the membrane of the palate and pharynx, were perceptible. There was no hoarseness, and scarcely any degree of difficulty of breathing. No essential relief had been obtained either by the bleeding or purging. It was observed, on the succeeding day, that the swelling and tenderness of the throat had augmented, but the febrile symptoms continued nearly the same. During the night the difficulty of deglutition increased, and the breathing became for the first time impeded. She was bled from the arm the following morning to sixteen ounces, and the blood appeared buffy, but not cupped. The relief afforded by this measure was, however, very trifling. At this period I was favoured with the assistance of my friend and colleague, Mr. Jeffreys, and we had soon an opportunity of observing the rapid strides with which the disease advanced. Mucus began to collect in large quantity about the glottis, and its expectoration occasioned extreme pain. The difficulty of breathing increased to so great a degree, that the tongue assumed a blue colour. To relieve this, blood was twice drawn from the arm, but the alleviation was very momentary. The blood, when drawn, had a very dark appearance. The difficulty of swallowing became speedily so great, as to preclude all possibility of administering remedies by the mouth. Leeches and fomentations were had recourse to. Latterly the patient complained of very considerable pain referred to the top of the sternum. After a great deal of suffering, she died on Monday, February 19th, seven days from the invasion of the disease.

The body was opened on the following day, in the presence of Mr. Jeffreys and a number of other gentlemen, whom the singularity of the case had attracted; and the appearances which presented themselves were these:—

The cellular membrane beneath the skin of the throat and around the trachea, as well as that which connects the pharynx and palate to the surrounding bones, was every where in a state of disease,—doubtless the result of inflammatory action. In some places, actual *sphacelus* had occurred: in others, it was in a state of what might be called *imperfect suppuration*. In one or two points, purulent matter was distinctly to be traced. The same disorganized condition of the cellular membrane pervaded the whole extent of the anterior mediastinum, even as low as the point of the ensiform cartilage.

While such was the state of the cellular membrane of the throat, the mucous expansions of the palate, pharynx, œsophagus, and trachea, were healthy, except in so far as they were covered with a preternaturally abundant secretion of mucus. The lungs and the different abdominal viscera were found free from any traces of disease.

To this singular variety of quinsy I have ventured to apply the term *cynanche cellularis*, from a belief that it has not yet received any more appropriate appellation. An extensive acquaintance with the works of the old authors might possibly have furnished me with cases offering an exact parallel to the one now detailed, but hitherto I have only succeeded in detecting one or two, which appear to resemble it in some of its characters.

Mr. James, of Exeter, in his late valuable work on Inflammation, describes a disease, under the title of "Angina Externa," which exhibits the following symptoms:—

"The patient," he says, "(perhaps a female,) of unhealthy and generally full and gross habit, has a swelling deep seated in the side of the neck, towards the angle of the jaw, causing a great degree of pain in that side of the head, from its effects upon the nerves of the part most probably, and accompanied with much pyrexia.

"There is loading of the cellular membrane, similar to that which we observe in erysipelas phlegmonodes, but well limited, firmer, and more prominent. This takes place to a great degree, and the result is that the patient is scarcely able to swallow fluids, breathes with great difficulty, and cannot sleep from the impending suffocation. After a time, the skin adheres and inflames, and thickens as it inflames, but does not point, or for a long time show any symptoms of pointing, or giving way by slough or ulceration: meanwhile sloughs and noisome pus have formed underneath, and do great mischief."*

Dr. Kirkland had previously described the same variety of cellular inflammation, and applied to it the name by which Mr. James designates it, Angina externa. As the case which he relates approaches more nearly than any I have yet met with to the subject of this paper, I may perhaps be permitted to refer to it.†

"A gentleman had a swelling with inflammation near the edge of the lower jaw, on the right side of the throat, *which soon extended itself to the other side*, and became so great that we had just reason to apprehend his being suffocated, if it continued to increase in size much longer; for it had already affected his breathing violently, and *he was almost choked with phlegm*, from pressure upon the windpipe. It had the appearance of suppurating, and a small quantity of matter seemed to fluctuate under the teguments, but so very deep that we durst not think of cutting into the side of the throat, where it was perceived, on account of the carotid artery. However, as no time was to be lost, I was determined, if possible, to make a drain from the part; for which purpose I made an incision at the lower part of the tumor, in the middle betwixt the sterno-hyoides, and was fortunate enough to reach the matter that had formed. Its gradual discharge put the patient out of all sort of danger, but the hardness was troublesome, and long in being subdued."

The only other case which I have met with resembling the one now detailed, is to be found in the third volume of the

* James *on Inflammation*, p. 188.

† See Kirkland's *Enquiry into the present State of Medical Surgery*, vol. ii. p. 159.

Transactions of the Society for the Improvement of Medical and Chirurgical Knowledge, p. 360. A case is there described, by Dr. Wells, of extensive gangrene of the cellular membrane between the muscles and skin of the neck and chest. In its early stage the disease was considered to be erysipelas, but it was remarkable for the great degree of swelling that attended it. Upon dissection, the cellular membrane of the neck and chest was found in a highly gangrenous state; but, as in the instance before us, this disorganized state of the cellular membrane was not communicated either to the skin or to the subjacent muscles.

This comprises all that I have been able to pick up from authors in illustration of the case which I have related. It will be perceived from hence that there is probably something very peculiar in its character; but, as I before stated, there may be several cases of a similar kind on record, and it will afford me great pleasure to have my attention directed to them, or to receive further information on the subject, from any of the numerous correspondents of this Journal.

98, *Great Portland-street; August* 1, 1822.

